# Roles of ASIC3, TRPV1, and Na_V_1.8 in the transition from acute to chronic pain in a mouse model of fibromyalgia

**DOI:** 10.1186/1744-8069-10-40

**Published:** 2014-06-23

**Authors:** Wei-Nan Chen, Cheng-Han Lee, Shing-Hong Lin, Chia-Wen Wong, Wei-Hsin Sun, John N Wood, Chih-Cheng Chen

**Affiliations:** 1 Graduate Institute of Life Sciences, National Defense Medical Center, Taipei 114, Taiwan; 2 Institute of Biomedical Sciences, Academia Sinica, 128 Academia Road, Section 2, Taipei 115, Taiwan; 3 Department of Life Sciences, National Central University, Jhongli 32054, Taiwan; 4 Wolfson Institute for Biomedical Research, Molecular Nociception Group, University College London, London WC1E 6BT, UK; 5 Taiwan Mouse Clinic—National Comprehensive Mouse Phenotyping and Drug Testing Center, Academia Sinica, 128 Academia Road, Section 2, Taipei 115, Taiwan

**Keywords:** Acidosis, APETx2, Hyperalgesic priming, IB4, PKCϵ

## Abstract

**Background:**

Tissue acidosis is effective in causing chronic muscle pain. However, how muscle nociceptors contribute to the transition from acute to chronic pain is largely unknown.

**Results:**

Here we showed that a single intramuscular acid injection induced a priming effect on muscle nociceptors of mice. The primed muscle nociceptors were plastic and permitted the development of long-lasting chronic hyperalgesia induced by a second acid insult. The plastic changes of muscle nociceptors were modality-specific and required the activation of acid-sensing ion channel 3 (ASIC3) or transient receptor potential cation channel V1 (TRPV1). Activation of ASIC3 was associated with increased activity of tetrodotoxin (TTX)-sensitive voltage-gated sodium channels but not protein kinase Cϵ (PKCϵ) in isolectin B4 (IB4)-negative muscle nociceptors. In contrast, increased activity of TTX-resistant voltage-gated sodium channels with ASIC3 or TRPV1 activation in Na_V_1.8-positive muscle nociceptors was required for the development of chronic hyperalgesia. Accordingly, compared to wild type mice, Na_V_1.8-null mice showed briefer acid-induced hyperalgesia (5 days vs. >27 days).

**Conclusion:**

ASIC3 activation may manifest a new type of nociceptor priming in IB4-negative muscle nociceptors. The activation of ASIC3 and TRPV1 as well as enhanced Na_V_1.8 activity are essential for the development of long-lasting hyperalgesia in acid-induced, chronic, widespread muscle pain.

## Background

Chronic muscle pain is a significant clinical problem affecting many people [1]. Although both peripheral and central sensitizations are believed involved in the transition from acute to chronic muscle pain, the underlying mechanism is not well understood [2-4]. An emerging hypothesis of hyperalgesic priming proposed by Jon Levine is that the transition might involve neural plasticity in primary afferent nociceptors, whereby an acute noxious stimulation can trigger long-lasting hypersensitivity of nociceptors to subsequent insults [5]. The hyperalgesic priming phenomenon occurs in a specific subset of nociceptors that bind isolectin B4 (IB4) and requires increased activity of tetrodotoxin (TTX)-resistant sodium channels and a switch in intracellular signaling pathways from protein kinase A to the epsilon isoform of protein kinase C (PKCϵ) in response to the same stimulus [6-8].

Tissue acidosis in muscles related to ischemia and inflammation has a profound effect on the initiation and development of chronic muscle pain [9,10]. Proton-sensing ion channels, such as acid-sensing ion channel 3 (ASIC3) and transient receptor potential cation channel V1 (TRPV1), are involved in activating muscle nociceptors and inducing the central sensitization that occurs in animal models of chronic muscle pain [11-13]. To probe how acid triggers chronic muscle pain, Sluka and colleagues developed a rodent model of chronic muscle pain induced by acid saline injected twice, 5 days apart, to the gastrocnemius muscle (GM) on one side of mice or rats [14,15]. The first acid injection triggers a transient referred hyperalgesia in both hind paws that diminishes in 24 h. The second acid injection 5 days later to the same side induces long-lasting referred hyperalgesia. Furthermore, although the dual acid injections are unilateral in the same site, the hyperalgesic effects are bilateral and the induction of chronic widespread pain shifts the autonomic balance to sympathetic predominance and decreases baroreflex sensitivity, which are related to findings in humans with chronic widespread pain or fibromyalgia [16].

The requirement of dual acid injections to evoke chronic muscle pain implies a hyperalgesic priming of muscle nociceptors after the first acid injection. Although proton-sensing ion channels are implicated in the initiation of hyperalgesia induced by intramuscular injections, only ASIC3 was confirmed to play an essential and sufficient role in triggering the acid-induced chronic muscle pain [15,17]. However, how ASIC3 induces the hyperalgesic priming is not known and whether TRPV1 is involved in the acid-induced, chronic, widespread pain is not clear.

In this study, we aimed to reveal the molecular mechanism underlying the transition from acute to chronic muscle pain in the acid-induced, chronic, widespread pain model. We tested how ASIC3 and/or TRPV1 activation affects the duration of the hyperalgesic priming state and the chronic pain state induced by dual intramuscular acid insults.

## Results

### Neural subgroups of acid-sensitive muscle afferent DRG neurons

**Figure 1:**
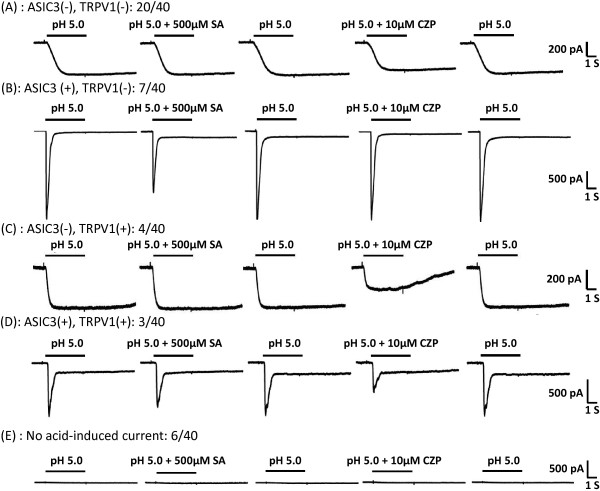
**Representative traces of 5 acid-evoked current types in small- to medium-sized muscle afferent dorsal root ganglion (DRG) neurons. (A)** In acid-sensing ion channel 3 (ASIC3)-negative, transient receptor potential cation channel V1 (TRPV1)-negative neurons, acid-induced currents were resistant to 500 μM salicylic acid (SA, a selective blocker for ASIC3) and 10 μM capsazepine (CZP, a selective blocker for TRPV1). **(B)** In ASIC3-positive, TRPV1-negative neurons, acid-induced currents were inhibited by SA but not by CZP. **(C)** In ASIC3-negative, TRPV1-positive neurons, acid-induced currents were inhibited by CZP but not by SA. **(D)** In ASIC3-positive, TRPV1-positive neurons, acid-induced currents were inhibited by both SA and CZP. **(E)** In a small subset of neurons, no acid-induced current was obtained. Numbers are number of neurons to total recorded neurons.

Although the expression of ASIC3 and its functional implications have been characterized in muscle nociceptors, the response of TRPV1-expressing muscle nociceptors to acid is poorly addressed. We first analyzed acid-induced currents in muscle afferent dorsal root ganglion (DRG) neurons and determined the neural populations of ASIC3- and TRPV1-expressing muscle nociceptors by inhibition with salicylic acid (SA, ASIC3 antagonist) and capsazepine (TRPV1 antagonist) [18,19]. Whole-cell patch clamp recording revealed that most of the small- to medium-sized (20–40 μm in diameter) muscle afferent DRG neurons expressed acid-induced inward currents (34/40), including 17.5% (7/40) ASIC3-like currents, 10% (4/40) TRPV1-like currents, and 7.5% (3/40) ASIC3-/TRPV1-like currents (Figure 1).

### Involvement of TRPV1 and ASIC3 in the establishment of hyperalgesic priming

**Figure 2:**
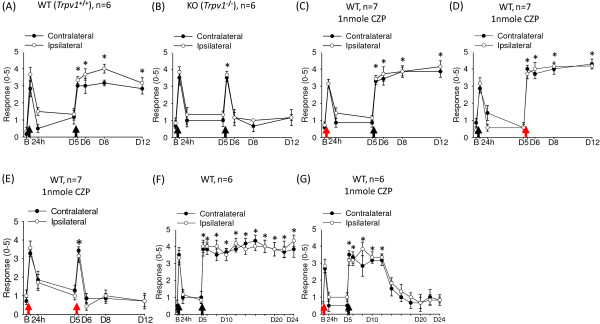
**Involvement of peripheral TRPV1 in intramuscular-acid–induced mechanical hyperalgesia.** The withdrawal responses of mouse hind paws to a 0.2-mN bending force in *Trpv1*^*+/+*^ and *Trpv1*^*−/−*^ mice before and after intramuscular acid injection. **(A)***Trpv1*^*+/+*^ and **(B)***Trpv1*^*−/−*^ mice were injected with pH 4.0 saline on days 0 and 5. **(C)** Co-injection of acid with capsazepine (1 nmole) at the first injection did not affect the development of hyperalgesia to the repeated acid injection in wild-type (WT) mice. **(D)** Capsazepine (1 nmole) at the second acid injection did not affect the development of hyperalgesia. **(E)** Capsazepine (1 nmole) at both acid injections prevented the development of long-lasting hyperalgesia. **(F)** Dual acid injections induced long-lasting hyperalgesia more than 19 days. **(G)** Coinjection of acid with capsazepine (1 nmole) at day 0 resulted in short-lasting hyperalgesia, for 7 days. Black arrows indicate when mice received the intramuscular acid injection. Red arrows indicate when mice received the co-injection of acid with capsazepine. B, baseline on day 0; D, day. *P < 0.05 compared with the response before the second acid injection.

We next examined whether deletion or inhibition of TRPV1 would affect the intramuscular-acid–induced hyperalgesia. *Trpv1*^*−/−*^ mice showed transient hyperalgesia after the first and second acid injections spaced 5 days apart but failed to show long-lasting hyperalgesia after the second acid injection as did *Trpv1*^*+/+*^ mice (Figure 2A and B). Interestingly, inhibiting TRPV1 by co-injection of acid and capsazepine at the first injection or at the second injection did not affect the development of long-lasting hyperalgesia (Figure 2C and D). Only co-injection of acid with capsazepine in both injections abolished the development of long-lasting hyperalgesia and produced an exact phenocopy of the *Trpv1* gene deletion (Figure 2E). These results suggest a role for TRPV1 in mediating the hyperalgesic priming and hypersensitivity of primed nociceptors. Although TRPV1 inhibition at the first acid injection did not abolish the hyperalgesic priming, it shortened the duration of the long-lasting hyperalgesia induced by the second acid-alone injection (Figure 2F and G). TRPV1 activation at the first acid injection may be required for establishing nociceptor priming, which is important for maintaining long-lasting hyperalgesia induced by a second acid insult.

**Figure 3:**
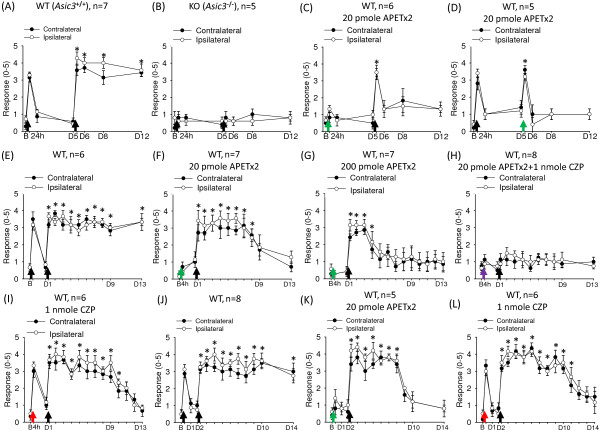
**Contribution of ASIC3 and TRPV1 to hyperalgesic priming in muscle nociceptors. (A,B)** Dual intramuscular acid injections induced chronic hyperalgesia in *Asic3*^*+/+*^ mice but did not induce hyperalgesia in *Asic3*^*−/−*^ mice. **(C)** Co-injection of acid with APETx2 (20 pmole) abolished the acid-induced transient hyperalgesia and prevented the development of long-lasting hyperalgesia with the second acid injection on day 5 in wild-type mice. **(D)** APETx2 (20 pmole) at the second acid injection produced only transient hyperalgesia in wild-type mice. **(E-I)** Mice received dual acid injections 1 day apart. The hyperalgesia lasted more than 12 days **(E)**. Mice developed shorter terms of hyperalgesia up to 7 or 3 days with the first acid injection combined with 20 pmole **(F)** or 200 pmole **(G)** APETx2, respectively. **(H)** Co-injection of 20 pmole APETx2 and 1 nmole capsazepine in the first acid injection abolished the development of long-lasting hyperalgesia with the second acid injection. **(I)** Co-injection of acid and 1 nmole capsazepine shortened the second acid-induced hyperalgesia to 9 days. **(J-L)** Mice received dual acid injections 2 days apart. No coinjection **(J)**, co-injection of acid and 20 pmole APETx2 **(K)**, and co-injection of acid and 1 nmole capsazepine **(L)** had different effects on hyperalgesia duration induced by the second acid injection. Black arrows indicate when mice received intramuscular acid injections. Green, red, and purple arrows indicate when mice received the co-injection of acid with APETx2, capsazepine, and APETx2 combined with capsazepine respectively. B, baseline on day 0; D, day. *P < 0.05 compared with the response before the second acid injection.

In contrast, *Asic3*^*−/−*^ mice showed neither transient nor long-lasting hyperalgesia with dual intramuscular acid injections spaced 5 days apart (Figure 3A, B). We next used a pharmacological approach to probe the role of ASIC3 in the hyperalgesic priming. With co-injection of acid with APETx2 (2 or 20 pmole), a selective ASIC3 antagonist [20], at the first injection, the transient hyperalgesia was not evoked; a second acid injection on day 5 evoked only transient hyperalgesia, which suggests that the nociceptors were in an unprimed state (Figure 3C). Co-injection of acid with APETx2 (20 pmole) at the second injection induced transient but not long-lasting hyperalgesia (Figure 3D). Therefore, the primed nociceptors require ASIC3 activation for developing chronic hyperalgesia in the dual acid-injection scheme.

### The duration of hyperalgesic priming

Although we did not observe nociceptor priming on day 5 when APETx2 inhibited ASIC3 at the first acid injection, we cannot exclude that a shorter duration of hyperalgesic priming was evoked with TRPV1. Thus, we tested whether activating TRPV1 only (by inhibiting ASIC3 with APETx2) could still contribute to short-term hyperalgesic priming in muscle nociceptors, if the dual acid injections were administered less than 5 days apart. With the dual acid injections administered 1 day apart, the second acid injection produced a robust long-lasting hyperalgesia for more than 12 days as compared with the basal responses before the second injection; however, the hyperalgesia lasted for 7 days with co-injection of acid with APETx2 (20 pmole) at the first injection (Figure 3E and F). The next-day acid-injection–induced hyperalgesia could still last for 3 days even with a higher dose of APETx2 (200 pmole) (Figure 3G). Interestingly, with co-injection of acid and APETx2 (20 pmole) and capsazepine, the response was totally blunted to the next-day acid injection (Figure 3H). Thus, TRPV1 and ASIC3 are the major proton-sensing ion channels in muscle nociceptors responsible for acid-induced hyperalgesic priming and hyperalgesia. TRPV1 could play a central role together with ASIC3 in the acid-induced hyperalgesic priming. We further validated this concept by finding that co-injection of acid with capsazepine shortened the duration of the long-lasting hyperalgesia induced by the second acid injection (Figure 3I).

We further tested the contribution of activating only TRPV1 or ASIC3 in the dual acid injections spaced 2 days apart (Figure 3J-L), and the results were very similar to the dual acid injections spaced 1 day apart. Thus, both ASIC3 and TRPV1 channels may contribute to the acid-induced hyperalgesic priming of muscle nociceptors, but both have a different contribution to the duration of the first acid injection-induced nociceptor priming and the maintenance of long-lasting hyperalgesia induced by the second acid injection (Table 1).

**Table 1:** Effect of acid-sensing ion channel 3 (ASIC3) and transient receptor potential cation channel V1 (TRPV1) inhibition on maintenance of hyperalgesia induced by dual acid injections

**First injection**	**Maintenance of hyperalgesia induced by the second acid injection on**		
	**Day 1**	**Day 2**	**Day 5**
Acid with APETx2	7 days (20 pmole)	6 days (20 pmole)	4 hr (20 pmole)
	3 days (200 pmole)		
Acid with capsazepine	9 days	9 days	7 days
Acid with vehicle	>12 days	>12 days	>12 days

### ASIC3 and TRPV1 activation enhanced TTX-sensitive (TTXs) and TTX-resistant (TTXr) voltage-gated sodium current (*I*_NaV_) in muscle nociceptors

**Figure 4:**
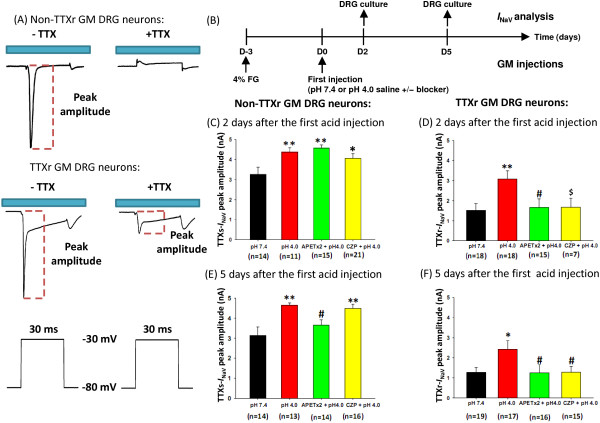
**The effect of ASIC3 and TRPV1 signaling on voltage-gated sodium currents (*I***_**NaV**_**) in the acid-induced muscle pain model. (A)** Representative current traces show tetrodotoxin (TTX)-sensitive and -resistant (TTXr) *I*_NaV_ evoked in medium-sized gastrocnemius muscle (GM) DRG neurons. **(B)** The experimental design of *I*_NaV_ analysis on muscle afferent DRG neurons. Mice were injected with 20 μL pH 7.4 saline, pH 4.0 saline, pH 4.0 saline with 20 pmole APETx2, or pH 4.0 saline with 1 nmole capsazepine. Effect of intramuscular acid injections on *I*_NaV_ in **(C)** non-TTXr and **(D)** TTXr GM DRG neurons isolated 2 days after acid injection. Effect of intramuscular acid injection on *I*_NaV_ in **(E)** non-TTXr and **(F)** TTXr GM DRG neurons isolated 5 days after acid injection. Data are mean ± SEM; *P < 0.05 and **P < 0.01 vs. pH 7.4; #P < 0.05 vs. pH4.0; $ P = 0.053 vs. pH4.0.

We next probed whether the ASIC3- and TRPV1-mediated hyperalgesic priming in muscle nociceptors resulted from *I*_NaV_ as seen in inflammation-induced hyperalgesic priming [7]. We analyzed the peak amplitudes of *I*_NaV_ in 2 types of muscle afferent DRG neurons, TTXr and non-TTXr neurons, on the basis of existence of a TTXr current (Figure 4A). We thus compared the *I*_NaV_ in muscle afferent DRG neurons at 2 or 5 days after an intramuscular injection of pH 7.4 saline or acid (pH 4.0 saline) with or without APETx2 or capsazepine (Figure 4B). As compared with pH 7.4 saline injection, at 2 days after acid injection, non-TTXr GM DRG neurons showed significantly enhanced TTXs *I*_NaV_. However, co-injection of acid and APETx2 or capsazepine did not reverse the acid-induced increase in TTXs *I*_NaV_ (Figure 4C). In contrast, although TTXr GM DRG neurons did not differ in the TTXs component of *I*_NaV_ with treatment, TTXr *I*_NaV_ was significantly increased with acid injection, and inhibition of ASIC3 or TRPV1 effectively reversed the acid-induced effect (Figure 4D). In non-TTXr GM DRG neurons, enhanced TTXs *I*_NaV_ was maintained with acid only or co-injection with capsazepine but not co-injection with APETx2 5 days after acid injection (Figure 4E), which suggests that inhibition of ASIC3 would shorten the duration of hyperalgesic priming. In TTXr GM DRG neurons, the enhanced TTXr *I*_NaV_ was still maintained with acid-only treatment 5 days after acid injection (Figure 4F). Taken together, the acid-induced plastic changes of nociceptors occurred in both non-TTXr and TTXr GM DRG neurons, with a significant increase of *I*_NaV_ in both muscle nociceptor populations.

### Subtypes of ASIC3-expressing muscle nociceptors

**Figure 5:**
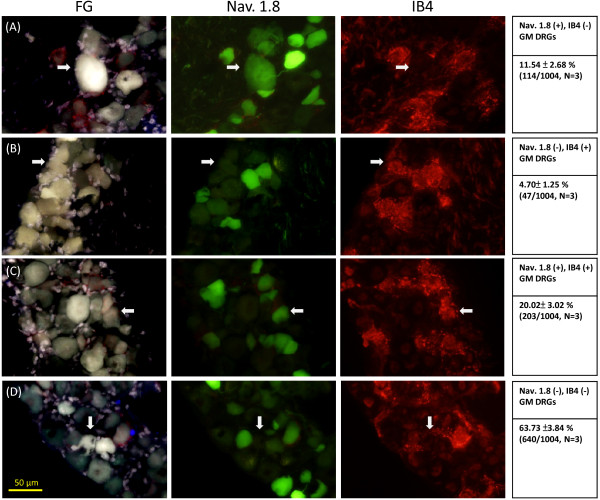
**Expression of Na**_**V**_**1.8 and isolectin B4 (IB4) in muscle afferent DRG neurons labeled with retrograde tracing of flourogold in *Na***_***V***_***1.8***^***+/−***^**-Cre mice carrying the CAG-STOP**^**floxed**^**-EGFP allele. (A)** Na_V_1.8-positive, IB4-negative neurons represented 11.5% of total muscle afferent DRG neurons. **(B)** Na_V_1.8-negtive, IB4-positive neurons represented 4.7% of total muscle afferent DRG neurons. **(C)** Na_V_1.8-positive, IB4-positive neurons represented 20.0% total muscle afferent DRG neurons. **(D)** Na_V_1.8-negative, IB4-negative neurons represented 63.7% of total muscle afferent DRG neurons. Arrows indicate the neurons of the cell type described at the right panel.

To better understand the cell-type–specific effects of ASIC3 and TRPV1 activation on TTXr *I*_NaV_, we used genetic tools to analyze how these proton-sensing ion channels related to Na_V_1.8 expression and whether they were expressed in IB4-positive neurons that are essential for hyperalgesic priming. We first examined the co-expression of Na_V_1.8 and IB4 in muscle afferent DRG neurons from mice that carry a Na_V_1.8-Cre allele to drive the GFP reporter allele (Figure 5). Among 1,004 muscle afferent DRG neurons (from 3 mice), 317 (31.6%) were Na_V_1.8-positive. Among the Na_V_1.8-positive muscle afferent DRG neurons, 64% (203/317) were co-localized with IB4, and the other 36% (114/317) were not. Because Na_V_1.8 contributes the most to TTXr *I*_NaV_ in DRG neurons, we next analyzed the acid-induced currents in Na_V_1.8-expressing GM DRG neurons. Before electrophysiological recordings, cultured DRG neurons were stained with IB4-DyLight to determine whether the Na_V_1.8-expressing GM DRG neurons were IB4-positive or -negative. We categorized the muscle afferent DRG neurons into 2 cell-sized groups, small-sized (20–30 μm in diameter) and medium-sized (30–40 μm in diameter), because we found all IB4-positive GM DRG neurons were smaller than 30 μm in diameter. Whole-cell patch clamp recording revealed that ASIC3 was expressed in 30% (9/30) of Na_V_1.8-positive and IB4-negative medium-sized GM DRG neurons but not in IB4-positive small-sized GM DRG neurons (Table 2). This result echoes our previous finding of acid-induced enhanced TTXr-*I*_NaV_ found only in medium-sized GM DRG neurons but not in small-sized GM DRG neurons [21]. We thus further examined the Na_V_1.8-negative medium-sized GM DRG neurons and found that 23% (6/26) expressed an ASIC3-like current. Interestingly, a high frequency of ASIC3-expressing neurons also expressed TRPV1 (Table 2). The expression of ASIC3 in both Na_V_1.8-positive and -negative medium-sized GM DRG neurons supports that ASIC3 activation could contribute to the acid-enhanced *I*_NaV_ in both TTXr and non-TTXr GM DRG neurons and that APETx2 could significantly attenuate the acid-enhanced *I*_NaV_.

**Table 2:** Electrophysiological characterization of ASIC3 and TRPV1 expression in subsets of muscle afferent dorsal root ganglion neurons

	**No. of acid-sensitive neurons in subsets of muscle afferent DRG neurons**		
**Type of acid-induced currents**	**Na**_**V**_**1.8(−), IB4(−)**	**Na**_**V**_**1.8(+), IB4(−)**	**Na**_**V**_**1.8(+), IB4(+)**
	**(30–40 μm)**	**(30–40 μm)**	**(<30 μm)**
ASIC3(+), TRPV1(−)	1 (4%)	4 (13%)	0
ASIC3(+), TRPV1(+)	5 (19%)	5 (17%)	0
ASIC3(−), TRPV1(+)	5 (19%)	2 (7%)	10 (40%)
ASIC3(−), TRPV1(−)	13 (50%)	14 (47%)	6 (24%)
No current	2 (8%)	5 (17%)	9 (36%)
**Total**	**26**	**30**	**25**

### Roles of Na_V_1.8 and PKCϵ in acid-induced chronic hyperalgesia

**Figure 6:**
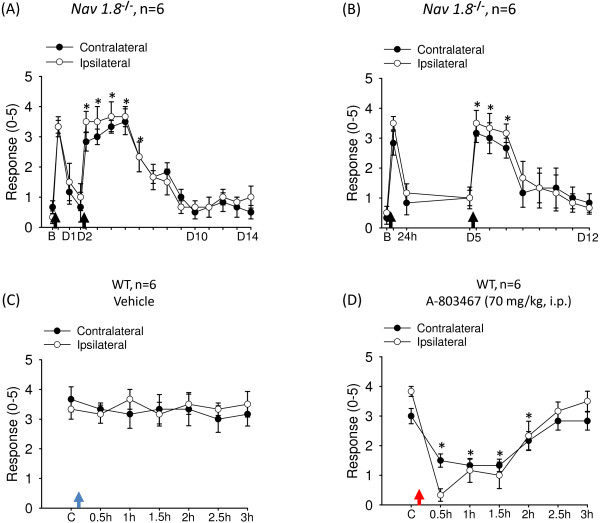
**Involvement of Na**_**V**_**1.8 in acid-induced chronic muscle pain. (A, B)** Dual acid injections spaced 2 **(A)** or 5 **(B)** days apart induced short-term hyperalgesia (2–4 days) in *Na*_*V*_*1.8*^*−/−*^ mice. **(C, D)** Analgesic effect of Na_V_1.8-selective blocker A-803467 was tested at 3 days after mice have developed chronic hyperalgesia induced by dual intramuscular acid injection spaced 5 days apart. The A-803467 (70 mg/kg, i.p.) or vehicle was injected immediately after the baseline response (control) had been obtained. B, baseline on day 0; D, day. Blue and red arrows indicate the time mice receive intraperitoneal injection of vehicle or A-803467 respectively. *P < 0.05 compared with the response before the second acid injection or control.

Although enhanced TTXr *I*_NaV_ was observed in PKCϵ-dependent nociceptor priming [7], we found no association of acid-induced nociceptor priming and ASIC3- or TRPV1-enhanced TTXr *I*_NaV_ (Figures 3 and 4). To determine the biological meaning of the acid-enhanced TTXr *I*_NaV_, we probed the effect of Na_V_1.8 deletion on the intramuscular acid-induced hyperalgesia. With dual acid injections spaced 2 or 5 days apart, *Na*_*V*_*1.8*^*−/−*^ mice showed transient hyperalgesia after the first acid injection but not long-lasting hyperalgesia after the second acid injection (Figure 6A, B). The second acid injection induced hyperalgesia that lasted for only 2 to 4 days in *Na*_*V*_*1.8*^*−/−*^ mice. These data suggest a role for Na_V_1.8 in establishing nociceptor priming, which is important for maintaining the long-lasting hyperalgesia induced by repeat acid injections, whereas the plastic changes of Na_V_1.8 did not contribute to setting the duration of priming. Furthermore, an Na_V_1.8-selecitve blocker, A-803467, had analgesic effects on wild-type mice that had developed chronic hyperalgesia induced by second acid injection, which suggests that Na_V_1.8 is involved in maintaining the acid-induced chronic hyperalgesia (Figure 6C, D).

**Figure 7:**
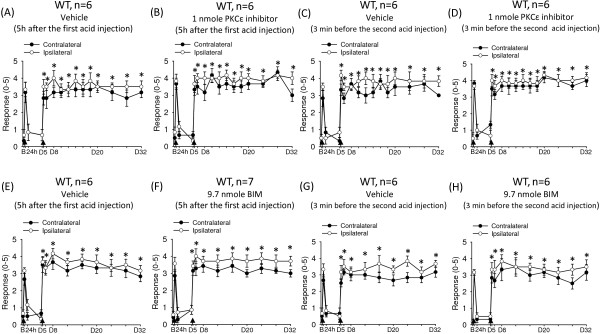
**Protein kinase Cϵ (PKCϵ) does not contribute to nociceptor priming in acid-induced muscle pain model. (A-D)** Effect of PKCϵ inhibitor peptide (V_1–2_, EAVSLKPT) on acid-induced hyperalgesia. Intramuscular injection of neutral saline **(A, C)** or 1 nmole PKCϵ inhibitor peptide **(B, D)** 5 hr after the first acid injection **(A, B)** or 3 min before the second acid injection **(C, D)** did not affect the development of hyperalgesia. **(E-H)** Effect of a general PKC inhibitor (BIM) on acid-induced hyperalgesia. Intramuscular injection of neutral saline **(E, G)** or 9.7 nmole BIM **(F, H)** 5 hr after the first acid injection **(E, F)** or 3 min before the second acid injection **(G, H)** did not affect the development of hyperalgesia. Black arrows indicate when mice received the intramuscular acid injection. B, baseline on day 0; D, day. *P < 0.05 compared with the response before the second acid injection.

Finally, we examined whether the acid-induced hyperalgesic priming depends on the activation of PKCϵ as seen in inflammatory pain models [5]. With the dual acid-injection model, we intramuscularly injected mice with the cell-permeable PKCϵ inhibitor peptide (TAT-PKCϵI) at 5 h after the first acid injection (Figure 7A, B) or 3 min before the second acid injection (Figure 7C, D). In both cases, the inhibition of PKCϵ activity did not affect the acid-induced long-lasting hyperalgesia. To further validate that PKCϵ (or other PKC isoforms) is not involved in the acid-induced hyperalgesic priming, we intramuscularly injected mice with a general PKC inhibitor, BIM, at 5 h after the first acid injection or 3 min before the second acid injection. Again, BIM had no effect on the acid-induced long-lasting hyperalgesia (Figure 7E-H). Therefore, activation of PKCϵ is not required in the acid-induced hyperalgesic priming.

## Discussion

### Acid-induced hyperalgesic priming is PKCϵ-independent in muscle nociceptors

**Figure 8:**
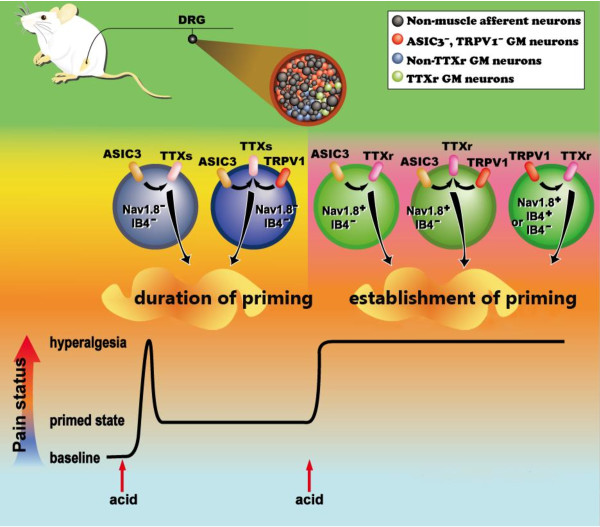
**A schematic model of ion channel-mediated hyperalgesic priming in muscle nociceptors.** ASIC3 and TRPV1 are expressed in different subsets of muscle nociceptors with or without Na_V_1.8 expression. In Na_V_1.8-negative muscle nociceptors, ASIC3 is the major acid sensor responsible for acid-induced transient hyperalgesia and the duration of hyperalgesic priming; TRPV1 may play a minor but essential role in the nociceptor priming. In Na_V_1.8-positive muscle nociceptors, both ASIC3 and TRPV1 contribute to the acid-enhanced TTXr *I*_NaV_, which is required for the establishment of priming that permits the development and maintenance of long-term hyperalgesia induced by a second acid insult. ASIC3 and TRPV1 are expressed alone or together, but ASIC3 is exclusively expressed in non-IB4 muscle nociceptors.

Accumulating evidence has suggested that the hyperalgesic priming of nociceptors is essential for the transition from acute to chronic pain states in many perplexing chronic pain conditions that are stress-related or neuropathic [5,22,23]. In inflammatory pain models, the hyperalgesic priming occurs exclusively in IB4-positive primary afferent nociceptors and depends on a switch in intracellular signaling pathways from PKA to PKCϵ [6,8,24]. The PKCϵ-dependent hyperalgesic priming is also present in vibration-induced muscle pain and chemotherapy-induced neuropathic pain and thus may constitute a general cellular basis for nociceptor plasticity in chronic pain [22,23]. Here we systematically examined the effect of the modality of the noxious acid insult on the duration of the hyperalgesia priming and the development of chronic hyperalgesia with a fixed, second acid injection. ASIC3 and TRPV1 were the major proton sensors responsible for the acid-induced hyperalgesic priming in non-IB4 muscle nociceptors, which manifests a new type of hyperalgesic priming mediated by ion channels (but not by PKCϵ) in the non-inflammatory model of chronic muscle pain (Figure 8).

### Acid-induced muscle nociceptor priming is modality-dependent mediated via ASIC3 and/or TRPV1

ASIC3 is responsible for the acid-induced transient hyperalgesia and hyperalgesic priming, and the development of chronic hyperalgesia to repeat acid injections. TRPV1 plays a minor but important role in the acid-induced hyperalgesic priming and the development of chronic hyperalgesia (Figure 8). The inhibitory effects of APETx2 and capsazepine on *I*_NaV_ enhancement were consistent with their effects on the duration of hyperalgesic priming and the maintenance of the chronic hyperalgesia induced by the second acid injection in the dual acid-injection model. Of note, inhibiting ASIC3 abolished the enhanced *I*_NaV_ in non-TTXr GM DRG neurons on day 5 but not day 2, so the lack of *I*_NaV_ enhancement was associated with no chronic hyperalgesia induced by the second acid injection in mice receiving APETx2 5 days previous. In contrast, the TTXr GM DRG neurons seemed to have little role in setting the priming state for future acid insult. Instead, the enhanced TTXr *I*_NaV_ in GM DRG neurons was inhibited by both APETx2 and capsazepine, which was associated with the effects of both drugs on shortening the hyperalgesia phase induced by the second acid injection (Table 1). Taken together, acid-induced hyperalgesic priming seems to be modality-dependent. ASIC3- and TRPV1-mediated nociceptor priming has differential effects on the development and maintenance of chronic hyperalgesia induced by a repeated acid insult.

### The duration of priming and the establishment of priming are mechanistically different

Our current study addresses neural subgroups involved in the acid-induced nociceptor priming and also different aspects of priming: (1) the duration of priming that determines how long the primed state can stay and (2) the establishment of priming that determines how long the second acid injection-induced hyperalgesia can be maintained (Figure 8). Mechanistically, in Na_V_1.8-negative muscle nociceptors, the activation of ASIC3 contributes to acid-induced transient hyperalgesia and enhanced TTX-s *I*_NaV_ as well as the duration of priming; TRPV1 might play a minor but essential role in setting the duration of priming. In contrast, in Na_V_1.8-positive muscle nociceptors, both ASIC3 and TRPV1 contribute to the acid-enhanced TTXr *I*_NaV_, which is required for the establishment of priming that permits the development and maintenance of long-term hyperalgesia induced by a second acid insult. Accordingly, although mice lacking Na_V_1.8 still showed hyperalgesic priming, they could develop hyperalgesia for only 2 to 4 days in the dual acid-injection model (Figure 6).

### ASIC3, TRPV1, and Na_V_1.8 play different roles in muscle pain associated with acidosis

Accumulating evidence has revealed that most metabo-nociceptive muscle afferent neurons contain ASIC3 and/or TRPV1, so both channels might be responsible for the muscle pain associated with acidosis [11,12,25]. Our study echoes this finding and further suggests a role for ASIC3 and TRPV1 in acid-induced hyperalgesic priming in muscle nociceptors. Although ASIC3 plays an important role in hyperalgesic priming and triggering chronic hyperalgesia, a recent study showed that ASIC3 is not involved in maintaining hyperalgesia in the dual acid-injection model [26]. Accordingly, the ASIC3-selective antagonists (e.g., APETx2) work in pre-emptive analgesia (a treatment that is initiated before injury or noxious stimulation to reduce sensitization) in rodent models of acid-induced chronic widespread pain, postoperative pain, and inflammatory pain but not in animals with chronic pain [27-30].

However, the role of TRPV1 in maintaining the acid-induced chronic hyperalgesia is still not known. Recent studies revealed that TRPV1 plays a role in the development of heat hypersensitivity after muscle inflammation and contributes to delayed onset muscle soreness downstream of NGF and GDNF [31,32]. TRPV1 is a pronociceptive polymodal receptor sensing for vanilloid compounds (e.g., capsaicin), noxious heat (>43°C) and low pH (<5.9) and could act as the final substrate of multiple inflammatory mediators that operate via distinct intracellular signaling pathways such as PKC [33]. PKCϵ-mediated potentiation of TRPV1 in DRG neurons contributes to heat hyperalgesia in rats [34]. However, PKC signaling is probably not involved in acid-induced TRPV1 activation in muscle nociceptors.

In contrast, the involvement of TTXr sodium channels in maintaining chronic hyperalgesia sheds light on the clinical use of the channel blocker. Na_V_1.8 is clearly not involved in setting the duration of hyperalgesic priming. Instead, the increased TTXr sodium current after the first acid injection was related to the long-lasting hyperalgesia after the second acid injection. In mice lacking Na_V_1.8, dual acid-injection–induced hyperalgesia was shortened to 2 to 4 days as compared with more than 19 days in wild-type mice. Accordingly, the Na_V_1.8-selective antagonist (A-803467) and general sodium channel blockers such as mexiletine or lamotrigine (37.5 mg/kg, intraperitoneally) have analgesic effects on the acid-induced, chronic, widespread pain model [35]. Thus, a selective sodium channel blocker (e.g., A-803467) might be a good choice to treat chronic muscle pain associated with recurrent ischemic insults [36]. Since the expression of Na_V_1.8 is restricted to the peripheral nervous system, the selective antagonists would reduce the risk of side effects on the central nervous system [37,38].

One concern with the study may be the selectivity of APETx2; a recent study revealed that APETx2 inhibited Na_V_1.8 currents of DRG neurons with an IC_50_ of 2.6 μM *in vitro*[39]. In the current study, we used a total of 20 pmole APETx2 (in 20 μL acid saline with a concentration of 1 μM APETx2) to inhibit the acid-induced nociceptor priming; with this dose, APETx2 should mostly inhibit homomeric ASIC3 channels or partially heteromeric ASIC3 channels and had little inhibitory effect on Na_V_1.8 [20,39]. As well, we found that 2 pmole APETx2 (at the first acid injection) was enough to abolish the second acid injection (on day 5) inducing chronic hyperalgesia (data not shown), which further confirmed the involvement of ASIC3 (but not Nav1.8) in the nociceptor priming. Nevertheless, a more selective ASIC3 antagonist without an inhibitory effect on Na_V_1.8 will be helpful to clearly distinguish the roles of ASIC3 and Na_V_1.8 in the acid-induced nociceptor priming.

### Is other acid-induced signaling involved in the acid-induced hyperalgesic priming?

Apart from Na_V_1.8, other cellular signaling might be involved in initiating the hyperalgesia in the first few days after the second acid insult or in regulating the increased activity of Na_V_1.8. For instance, we previously showed that substance P-mediated antinociceptive signaling in muscle nociceptors is diminished after repeat acid injection [21]. Intramuscular acid stimulation triggers the release of substance P from muscle nociceptors, which acts on NK1 receptors and activates M-type potassium via a G-protein–independent but Src-kinase–dependent manner. As well, proton-sensing G-protein–coupled receptors (e.g., G2A, GPR4, OGR1, TDAG8) and MrgprB4 are abundantly expressed in ASIC3-positive nociceptors and may contribute to the development of the intramuscular acid-induced hyperalgesia [40-42]. Moreover, other acid-induced responses in muscle afferent DRG neurons express neither ASIC3 nor TRPV1 (Figure 1 and Table 2) [43-46]. Future studies of muscle nociceptor-specific acid signaling would bring new insights into the molecular mechanism of chronic muscle pain and new opportunities for effective treatment.

Recent studies show that IB4-positive muscle nociceptors are responsible for chronic muscle pain triggered by acute inflammation (e.g., intramuscular carrageenan or glial cell-derived neurotrophic factor) or ergonomic intervention (e.g., eccentric exercise or vibration) [47,48]. Although Na_V_1.8 is largely expressed in IB4-positive muscle nociceptors, ASIC3 is exclusively expressed in non-IB4 muscle afferent DRG neurons with or without Na_V_1.8 expression (Table 2). However, we cannot exclude the role of IB4-positive muscle nociceptors in the development of long-lasting hyperalgesia in the dual acid-injection model, because many IB4- and Na_V_1.8-positive muscle nociceptors express TRPV1, which also contributes to the enhanced TTXr *I*_NaV_ and the chronic hyperalgesia induced by the second acid injection. Because TRPV1 channels are expressed in both IB4-positive and -negative muscle nociceptors, further studies should explore the differential roles of these 2 TRPV1-expressing muscle nociceptors in the pathogenesis of acid-induced chronic widespread pain.

### Does the acid-induced priming effect occur in the central nervous system?

The priming hypothesis aims to describe a new mode of neuroplastic change in primary afferent nociceptors, in which basal nociceptive thresholds are still normal but nociceptors are sensitized against exposure to algogens or sensitizing agents [5]. However, whether the priming effect occurs in the central nervous system is not known. In the acid-induced chronic widespread pain model, the first acid injection induces a transient hyperalgesia that declines in 24 h (basal nociceptive threshold is back to normal in von Frey assay), but a priming effect lasts for 5 days, annotated by an increase in *I*_NaV_ in muscle nociceptors and a potential to develop chronic hyperalgesia in response to future acid insults. Although we have focused on the plastic changes of noccieptors in the primed state (1–5 days after the first acid injection), we cannot exclude possible plastic changes in the central nervous system after the first intramuscular acid injection.

The bilateral effect from a unilateral intramuscular injection suggests the involvement of central sensitization. Evidence has shown the unilateral dual acid injections induce activation of the cAMP pathway in the spinal cord, ERK activation in the anterior nucleus of paraventricular thalamus (PVA), and plastic changes in capsular central amygdaloid neurons [49-51]. However, no study has addressed whether a single acid injection can induce a plastic change (or the priming effect) in the central nervous system. We have not been able to demonstrate a plastic change in capsular central amygdaloid neurons in the primed state (Cheng SJ and Chen CC, unpublished observation). Interestingly, our previous studies showed that blockade of Ca_V_3.2 T-type Ca^2+^ channel signaling or ERK activation in the PVA at 15 min before the second acid injection can prevent the chronic hyperalgesia, which suggests a possible central role of the PVA in the primed state [50]. Furthermore, acid can only induce transient hyperalgesia but not chronic hyperalgesia in mice lacking Ca_V_3.2. Further studies of Ca_V_3.2–dependent synaptic plasticity of PVA circuits in the primed state may shed light on the central control of the transition from acute to chronic pain.

## Conclusions

In conclusion, our data manifest a new type of nociceptor priming mechanism that involves activation of ASIC3 and TRPV1 in muscle nociceptors and requires the development of acid-induced, chronic, widespread muscle pain. We highlight the role of Na_V_1.8 in developing and maintaining the chronic pain and rule out the involvement of IB4-positive nociceptors and PKCϵ signaling in the transition from acute to chronic pain. These results will be clinically useful, because we provide a new opportunity for mechanism-based treatment for chronic, widespread muscle pain resulting from re-current acid insults possibly associated with symptoms of fibromyalgia and myofascial pain syndrome [11,52].

## Methods

### Animals

We used adult (8- to 12-wk-old) male C57/BL6 mice. All procedures followed the US Guide for the Care and Use of Laboratory Animals (National Academy of Sciences, Washington DC) and were approved by the Institutional Animal Care and Use Committee of Academia Sinica. *Asic3*^*−/−*^ and *Na*_*V*_*1.8*^*−/−*^*-Cre* mice were generated and genotyped as described [53,54]. *Trpv1*^*−/−*^ mice were purchased from the Jackson Lab (Bar Harbor, ME). All null-mutant mice were backcrossed to C57BL6 mice for at least 10 generations to establish a congenic strain. Congenic *Asic3*^*−/−*^, *Na*_*V*_*1.8*^*−/−*^*-Cre*, and *Trpv1*^*−/−*^ mice were offspring of *Asic3*^*+/−*^, *Na*_*V*_*1.8*^*+/−*^*-Cre*, and *Trpv1*^*+/−*^ intercrosses respectively. To identify Na_V_1.8-positive dorsal root ganglion (DRG) neurons, *Na*_*V*_*1.8*^*−/−*^*-Cre* mice were crossed with mice carrying a CAG-CAT-enhanced green fluorescent protein (CAG-CAT-EGFP) reporter allele with a stop-floxed segment inserted upstream of the EGFP (CAG-STOP^floxed^-EGFP) [55].

### Behavioral assays

**Figure 9:**
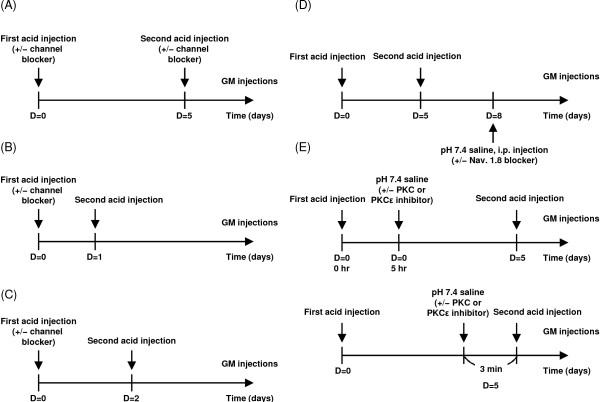
**The temporal sequence of five experimental groups in the dual acid injection model. (A)** Experimental design to probe the role of ASIC3 and TRPV1 in acid-induced hyperalgesia in the dual acid-injection scheme spaced 5 days apart. **(B,C)** Experimental design to test the role of ASIC3 and TRPV1 in acid-induced hyperalgesic priming when the second acid injection was given at day 1 **(B)** or day 2 **(C)**. **(D)** Experimental design to test the analgesic effect of Na_V_1.8 blocker on the acid-induced chronic widespread pain. **(E)** Experimental designs to test the role of PKCϵ on acid-induced hyperalgesic priming.

Mice received 2 injections, spaced 1–5 days apart, into the GM, of 20 μL acid saline (pH 4.0) with or without capsazepine (1 nmole), APETx2 (2, 20, or 200 pmole), or capsazepine (1 nmole) with APETx2 (20 pmole) (Figure 9A-C). The acid saline was prepared in 10 mM 2-[N-morpholino]ethanesulfonic acid and adjusted to pH 4.0 with 1 N NaOH. To test the effect of Na_V_1.8 on the maintenance of chronic hyperalgesia, the selective channel blocker A-803467 (Tocris, Avonmouth, UK) was dosed in 70 mg/kg intraperitoneally at 3 days after the mice have received dual acid injection (Figure 9D). To test the effect of PKCϵ, the PKCϵ inhibitor peptide (V_1–2_, EAVSLKPT) was conjugated with protein transduction domain of TAT protein (CYGRKKRRQRRR-CEAVSLKPT, TAT-PKCϵI) and was kindly provided from KAI pharmaceuticals (South San Francisco, CA). We injected 20 μL of the cell-permeable TAT-PKCϵI (50 μM in pH 7.4 saline) or a general PKC inhibitor BIM (485 μM in pH 7.4 saline, purchased from Cayman Chemical, Ann Arbor, Michigan) into the GM 5 h after the first acid injection or 3 min before the second injection (Figure 9E). Mechanical hyperalgesia was assessed as described [21]. Briefly, a 0.2 mN von Frey filament was applied to the plantar surface of both hind paws. A positive response was defined as foot lifting when the von Frey filament was applied. For each paw, the filament was applied 5 times at 30-s intervals. The experimenters were blinded to the experimental manipulations and/or mouse genotypes.

### DRG primary culture

To retrograde-trace muscle afferent DRG neurons, mice were anesthetized with 2% isoflurane and injected with 10 μL of 4% (wt/vol) fluorogold into the GM of both legs for 5 to 8 days. Lumbar DRG neurons were isolated, dissociated and cultured as described [21]. After seeding, DRG neurons were maintained in a 5% (vol/vol) CO_2_ incubator at 37°C and used for patch-clamp recording within 30 h. IB4-positive neurons were determined by staining with IB4-DyLight 594 (2.5 μg/mL in solution containing 0.1 mM MgCl_2_, CaCl_2_, and MnCl_2_; Vector Lab) for 2 min immediately before the recording. To visualize Na_V_1.8-expressing GM DRG neurons, DRG neurons were isolated from *Na*_*V*_*1.8*^*+/−*^-Cre mice carrying the CAG-STOP^floxed^-EGFP allele.

### Whole-cell patch-clamp recording

Whole-cell patch clamp recordings of muscle afferent DRG neurons involved use of an Axopatch MultiClamp 700B (Axon Instruments). Neurons with membrane potential > −40 mV were not accepted. The bridge was balanced in the current clamp mode and the series resistance was compensated 70% in voltage clamp mode with Axopatch 700B compensation circuitry. All DRG neuron recordings were performed at room temperature and were completed within 30 h after plating. The recording electrodes had a resistance of 1–5 MΩ when filled with an internal solution containing (in mM) 100 KCl, 2 Na_2_-ATP, 0.3 Na_3_-GTP, 10 EGTA, 5 MgCl_2_, and 40 Hepes, adjusted to pH 7.4 with KOH. Recording cells were superfused in artificial cerebrospinal fluid (ACSF) containing (in mM) 130 NaCl, 5 KCl, 1 MgCl_2_, 2 CaCl_2_, 10 glucose, and 20 Hepes, adjusted to pH 7.4 with NaOH. Osmolarity was adjusted to approximately 300 mOsm. ACSF was controlled by gravitational force. The acidic ACSF was titrated to pH 5.0 by 2-[N-morpholino]ethanesulfonic acid (MES). Salicylic acid (SA) was prepared from a 1-M stock solution (in 100% ethanol) to a final concentration of 500 μM in ACSF. Capsazepine was prepared from a 20 mM stock solution (in 100% ethanol) to a final concentration of 10 μM in ACSF. TTX was prepared from a 100-μM stock solution to a final concentration of 200 nM in ACSF. Capsazepine and TTX were purchased from Torcis (Avonmouth, UK) and APETx2 was from Alomone (Jerusalem, Israel). Otherwise, drugs were from Sigma Chemicals (St. Louis, MO).

### Acid-induced currents

First, a 1-ms 2 nA current step was used to evoke an action potential (AP). An AP with inflected falling phase indicates involvement of a TTXr sodium channel [56]. To determine whether TTXr sodium channels contributed to the AP configuration, inflections were determined by differentiation of AP. To obtain acid-induced currents, the acidic ACSF was applied through a glass pipette 50 μm from the neuron and via gravity controlled by a VC-6 six-channel valve controller (Warner Instruments). Acidic ACSF was applied for 4 s in 30-s intervals. After 3 applications, SA (500 μM) was bath-applied to examine whether the acid-induced current was inhibited. SA-containing bath was then replaced with normal ACSF for another 3 min. Next, capsazepine was bath-applied to examine whether the acid-induced current was inhibited. ASIC3-expressing neurons were defined when the acid-induced current was inhibited by SA [18]; TRPV1-expressing neurons were defined when the acid-induced current was inhibited by capsazepine [19].

### Voltage-gated sodium currents

Mice were injected first with 4% (wt/vol) fluorogold into GM and 3 days later with acidic saline (pH 4.0, 20 μL) alone or with 1 nmole capsazepine or 20 pmole APETx2. Mice were killed 2 or 5 days later, and DRG neurons were isolated and cultured as stated above and used to study voltage-gated sodium currents. Medium-sized DRG neurons with cell diameters 30–40 μm were selected for recording. The internal solution contained (in mM) 10 NaCl, 110 CsCl, 20 tetraethylammonium-chloride, 2.5 MgCl_2_, 5 EGTA, 3 Mg^2+^-ATP, and 5 Hepes, adjusted to pH 7.0 with CsOH. The external solution contained (in mM) 100 NaCl, 5 CsCl, 30 tetraethylammonium-chloride, 1.8 CaCl_2_, 1 MgCl_2_, 0.1 CdCl_2_, 25 glucose, 5 4-aminopyridine, and 5 Hepes, adjusted to pH 7.4 with HCl. Osmolarity was adjusted to 300 mOsm with glucose. The *I*_NaV_ was evoked by a 30-ms test pulse at −30 mV from a holding potential of −80 mV. For TTXr sodium currents, recordings were performed in external solution containing 200 nM TTX.

### Immunohistochemistry

To examine the expression of Na_V_1.8 and IB4 in muscle afferent DRG neurons, *Na*_*V*_*1.8*^+/−^-Cre mice carrying the CAG-STOP^floxed^-EGFP allele were injected with 4% (wt/vol) fluorogold into the GM 5 days before DRG isolation. Lumbar DRGs (L3-5) were isolated and fixed with 4% paraformaldehyde (in pH 7.4 phosphate buffered saline [PBS]) at 4°C for 2 h. Post-fixed tissues were placed in 20% sucrose at 4°C overnight, then embedded in OCT and rapidly frozen with use of dry ice and stored at −80°C. Frozen sections 12-μm thick were cut on a cryostat and mounted on glass slides. Slides were fixed with 4% paraformaldehyde at 4°C for 10 min, then incubated with blocking solution containing 1% bovine serum albumin, 0.1% Triton X-100, 0.02% sodium azide in PBS for 1 h at room temperature. After a PBS wash, the slides were stained with IB4-DyLight 594 for 30 min at room temperature.

### Data analysis

Data for *I*_NaV_ are presented as mean ± SEM and were analyzed by use of Origin 8.0 (OriginLab). One-way ANOVA and then Fisher’s least significant difference post-hoc test were used to compare differences between groups. The Mann–Whitney U test was used to compare withdrawal responses to von Frey filament application in mice before and after acid injection. P < 0.05 was considered statistically significant.

## Abbreviations

ASIC3: Acid-sensing ion channel 3; DRG: Dorsal root ganglion; GM: Gastrocnemius muscle; IB4: Isolectin B4; *I*_NaV_: Voltage-gated sodium current; Na_V_1.8: Voltage-gated sodium channel 1.8; PKCϵ: Protein kinase Cϵ; TRPV1: Transient receptor potential V1; TTX: Tetrodotoxin.

## Competing interest

The authors declare no competing financing interests.

## Authors’ contributions

WN Chen conducted and analyzed electrophysiological experiments. WN Chen, CH Lee, CW Wong performed behavioral experiments. WN Chen, SH Lin, WH Sun, CC Chen designed experiments. JN Wood provided Na_V_1.8 mice and interpreted data. CC Chen collected, integrated, and interpreted the results and wrote the manuscript. All authors read and approved the final manuscript.

## References

[B1] Mense S Muslce pain: mechanisms and clinical significance Dtsch Arztebl Int 2008 105 214 219 19629211 10.3238/artzebl.2008.0214 PMC2696782

[B2] Arendt-Nielsen L Fernandez-de-las-Penas C Graven-Nielsen T Basic aspects of musculoskeletal pain: from acute to chronic pain J Man Manip Ther 2011 19 186 193 23115471 10.1179/106698111X13129729551903 PMC3201649

[B3] DeSantana JM Sluka KA Central mechanisms in the maintenance of chronic widespread noninflammatory muscle pain Curr Pain Headache Rep 2008 12 338 343 18765138 10.1007/s11916-008-0057-7 PMC2744440

[B4] Staud R Peripheral pain mechanisms in chronic widespread pain Best Pract Res Clin Rheumat 2011 25 155 164 10.1016/j.berh.2010.01.010 PMC3220877 22094192

[B5] Reichling DB Levine JD Critical role of nociceptor plasticity in chronic pain Trend Neurosci 2009 32 611 618 19781793 10.1016/j.tins.2009.07.007 PMC2787756

[B6] Bogen O Alessandri-Harber N Chu C Gear RW Levine JD Generation of a pain memory in the primary afferent nociceptor triggered by PKCϵ activation of CPEB J Neurosci 2012 32 2018 2026 22323716 10.1523/JNEUROSCI.5138-11.2012 PMC3305286

[B7] Dina OA McCarter GC de Coupade C Levine JD Role of the sensory neuron cytoskeleton in second messenger signaling for inflammatory pain Neuron 2003 39 613 624 12925276 10.1016/s0896-6273(03)00473-2

[B8] Joseph EK Levine JD Hyperalgesic priming is restricted to isolectin B4-positive nociceptors Neuroscience 2010 169 431 435 20457222 10.1016/j.neuroscience.2010.04.082 PMC2903040

[B9] Frey Law LA Sluka KA McMullen T Lee J Arendt-Nielsen L Graven-Nielsen T Acidic buffer induced muscle pain evokes referred pain and mechanical hyperalgesia in humans Pain 2008 140 254 264 18835099 10.1016/j.pain.2008.08.014 PMC2613646

[B10] Issberner U Reeh PW Steen KH Pain due to tissue acidosis: a mechanism for inflammatory and ischemic myalgia? Neurosci Lett 1996 208 191 194 8733302 10.1016/0304-3940(96)12576-3

[B11] Birdsong WT Fierro L Williams FG Spelta V Naves LA Knowles M March-Haffner J Adelman JP Almers W Elde RP McCleskey EW Sensing muscle ischemia: coincident detection of acid and ATP via interplay of two ion channels Neuron 2010 68 739 749 21092862 10.1016/j.neuron.2010.09.029 PMC3000793

[B12] Fujii Y Ozaki N Taguchi T Mizumura K Furukawa K Sugiura Y TRP channels and ASICs mediate mechanical hyperalgesia in models of inflammatory muscle pain and delayed onset muscle soreness Pain 2008 140 292 304 18834667 10.1016/j.pain.2008.08.013

[B13] Hoheisel U Reinohl J Unger T Mense S Acidic pH and capsaicin activate mechanosensitive group IV muscle receptors in the rat Pain 2004 110 149 157 15275762 10.1016/j.pain.2004.03.043

[B14] Sluka KA Kalra A Moore SA Unilateral intramuscular injections of acidic saline produce a bilateral, long-lasting hyperalgesia Muscle Nerve 2001 24 37 46 11150964 10.1002/1097-4598(200101)24:1<37::aid-mus4>3.0.co;2-8

[B15] Sluka KA Price MP Breese NM Stucky CL Wemmie Welsh MJ Chronic hyperalgesia induced by repeated acid injections in muscle is abolished by the loss of ASIC3, but not ASIC1 Pain 2003 106 229 239 14659506 10.1016/S0304-3959(03)00269-0

[B16] Oliveira LR de Melo VU Macedo FN Barreto AS Badaue-Passos D Jr dos Santos MRV Dias DPM Sluka KA DeSantana JM Santana-Filho VJ Induction of chronic non-inflammatory widespread pain increases cardiac sympathetic modulation in rats Auton Neurosci 2012 167 45 49 22266357 10.1016/j.autneu.2011.12.004 PMC4667978

[B17] Yen YT Tu PH Chen CJ Lin YW Hsieh ST Chen CC Role of acid-sensing ion channel 3 in sub-acute-phase inflammation Mol Pain 2009 5 1 19126241 10.1186/1744-8069-5-1 PMC2632618

[B18] Lin YW Min MY Lin CC Chen WN Wu WL Yu HM Chen CC Identification and characterization of a subset of mouse sensory neurons that express acid-sensing ion channel 3 Neuroscience 2008 151 544 557 18082972 10.1016/j.neuroscience.2007.10.020

[B19] Tominaga M Caterina MJ Malmberg AB Rosen TA Gilbert H Skinner K Raumann RE Basbaum AI Julius D The cloned capsaicin receptor integrates multiple pain-producing stimuli Neuron 1998 21 531 543 9768840 10.1016/s0896-6273(00)80564-4

[B20] Diochot S Baron A Rash LD Deval E Escoubas P Scarzello S Salinas M Lazdunski M A new sea anemone peptide, APETx2, inhibits ASIC3, a major acid-sensitive channel in sensory neurons EMBO J 2004 23 1516 1525 15044953 10.1038/sj.emboj.7600177 PMC391081

[B21] Lin CCJ Chen WN Chen CJ Lin YW Zimmer A Chen CC An antinociceptive role for substance P in acid-induced chronic muscle pain Proc Natl Acad Sci USA 2012 109 E76 E83 22084095 10.1073/pnas.1108903108 PMC3258641

[B22] Alvarez P Ferrari LF Levine JD Muscle pain in models of chemotherapy-induced and alcohol-induced peripheral neuropathy Ann Neurol 2011 70 101 109 21786301 10.1002/ana.22382 PMC3145965

[B23] Dina OA Joseph EK Levine JD Green PG Mechanisms mediating vibration-induced chronic musculoskeletal pain analyzed in the rat J Pain 2010 11 369 377 19962353 10.1016/j.jpain.2009.08.007 PMC2847637

[B24] Ferrari LF Bogen O Levine JD Nociceptor subpopulations involved in hyperalgesic priming Neuroscience 2010 165 896 901 19931357 10.1016/j.neuroscience.2009.11.029 PMC2815163

[B25] Jankowski MP Rau KK Ekmann KM Anderson CE Koerber HR Comprehensive phenotyping of group III and iV muscle afferents in mouse J Neurophysiol 2013 109 2374 2381 23427306 10.1152/jn.01067.2012 PMC3652219

[B26] Gautam M Benson CJ Ranier JD Light AR Sluka KA ASICs do not play a role in maintaining hyperalgesia induced by repeated intramuscular acid injections Pain Res Treat 2012 2012 817347 22191025 10.1155/2012/817347 PMC3236358

[B27] Deval E Noel J Lay N Alloui A Diochot S Friend V Jodar M Lazdunski M Lingueglia E ASIC3, a sensor of acidic and primary inflammatory pain EMBO J 2008 27 3047 3055 18923424 10.1038/emboj.2008.213 PMC2585165

[B28] Deval E Noel J Gasull X Delaunay A Alloui A Friend V Eschalier A Lazdunski M Lingueglia E Acid-sensing ion channels in postoperative pain J Neurosci 2011 31 6059 66 21508231 10.1523/JNEUROSCI.5266-10.2011 PMC6632959

[B29] Karczewski J Spencer RH Garsky VM Liang A Leitl MD Cato MJ Cook SP Kane S Urban MO Reversal of acid-induced and inflammatory pain by the selective ASIC3 inhibitor, APETx2 Br J Pharmacol 2010 161 950 60 20860671 10.1111/j.1476-5381.2010.00918.x PMC2992907

[B30] Wu WL Cheng CF Sun WH Wong CW Chen CC Targeting ASIC3 for pain, anxiety, and insulin resistance Pharmacol Ther 2012 134 127 138 22233754 10.1016/j.pharmthera.2011.12.009

[B31] Ota H Katanosaka K Murase S Kashio M Tominaga M Mizumura K TRPV1 and TRPV4 play pivotal roles in delayed onset muscle soreness PLoS ONE 2013 8 e65751 23799042 10.1371/journal.pone.0065751 PMC3684597

[B32] Walder RY Radhakrishnan R Loo L Rasmussen LA Mohapatra DP Wilson SP Sluka KA TRPV1 is important for mechanical and heat sensitivity in uninjured animals and development of heat hypersensitivity after muscle inflammation Pain 2012 153 1664 1672 22694790 10.1016/j.pain.2012.04.034 PMC3494878

[B33] Levine JD Alessandri-Harber: TRP channels: targets for the relief of pain Biochim Biophys Acta 2007 1772 989 1003 17321113 10.1016/j.bbadis.2007.01.008

[B34] Zhang H Cang CL Kawasaki Y Liang LL Zhang YQ Ji RR Zhao ZQ Neurokinin-1 receptor enhances TRPV1 activity in primary sensory neurons via PKCϵ: a novel pathway for heat hyperalgesia J Neurosci 2007 27 12067 12077 17978048 10.1523/JNEUROSCI.0496-07.2007 PMC6673346

[B35] Nielsen AN Mathiesen C Blackburn-Munro G Pharmacological characterization of acid-induced muscle allodynia in rats Eur J Pharmacol 2004 487 93 103 15033380 10.1016/j.ejphar.2004.01.017

[B36] Jarvis MF Honore P Shieh CC Chapman M Joshi S Zhang XF Kort M Carroll W Marron B Atkinson R Thomas J Liu D Krambis M Liu Y McGaraughty S Chu K Roeloffs R Zhong C Mikusa JP Hernandez G Gauvin D Wade C Zhu C Pai M Scanio M Shi L Drizin I Gregg R Matulenko M Hakeem A Gross M Johnson M Marsh K Wagoner PK Sullivan JP Faltynek CR Krafte DS A-803467, a potent and selective Na_V_1.8 sodium channel blocker, attenuates neuropathic and inflammatory pain in the rat Proc Natl Acad Sci USA 2007 104 8520 5 17483457 10.1073/pnas.0611364104 PMC1895982

[B37] Akopian AN Sivilotti L Wood JN A tetrodotoxin-resistant voltage-gated sodium channel expressed by sensory neurons Nature 1996 379 257 62 8538791 10.1038/379257a0

[B38] Eijkelkamp N Linley JE Baker MD Minett MS Cregg R Werdehausen R Rugiero F Wood JN Neurological perspectives on voltage-gated sodium channels Brain 2012 135 2585 2612 22961543 10.1093/brain/aws225 PMC3437034

[B39] Blanchard MG Rash LD Kellenberger S Inhibition of voltage-gated Na + currents in sensory neurones by the sea anemone toxin APETx2 Br J Pharmacol 2012 165 2167 2177 21943094 10.1111/j.1476-5381.2011.01674.x PMC3413854

[B40] Huang CW Tzeng JN Chen YJ Tsai WF Chen CC Sun WH Nociceptors of dorsal root ganglion express proton-sensing G-protein-coupled receptors Mol Cell Neurosci 2007 36 195 210 17720533 10.1016/j.mcn.2007.06.010

[B41] Huang YH Chang CY Chen CC Yang CD Sun WH Distinct expression of Mas1-related G-protein-coupled receptor B4 in dorsal root and trigeminal ganglia-implications for altered behaviors in acid-sensing ion channel-deficient mice J Mol Neurosci 2013 51 820 834 23900719 10.1007/s12031-013-0070-0

[B42] Su YS Sun WH Chen CC Molecular mechanism of inflammatory pain World J Anesthesiol 2014 3 71 81

[B43] Chen CC Wong CW Neurosensory mechanotransduction through acid-sensing ion channels J Cell Mol Med 2013 17 337 349 23490035 10.1111/jcmm.12025 PMC3823015

[B44] Deval E Gasull X Noel J Salinas M Baron A Diochot S Lingueglia E Acid-sensing ion channels (ASICs): pharmacology and implication in pain Pharmacol Ther 2010 128 549 558 20807551 10.1016/j.pharmthera.2010.08.006

[B45] Gautam M Benson CJ Acid-sensing ion channels (ASICs) in mouse skeletal muscle afferents are heteromers composed of ASIC1a, ASIC2, and ASIC3 subunits FASEB J 2013 27 793 802 23109675 10.1096/fj.12-220400 PMC3545527

[B46] Chen WN Chen CC Acid mediates a prolonged antinociception via substance P signaling in acid-induced chronic widespread pain Mol Pain 2014 10 30 24886508 10.1186/1744-8069-10-30 PMC4039541

[B47] Alvarez P Chen X Bogen O Green PG Levine JD IB4(+) nociceptors mediate persistent muscle pain induced by GDNF J Neurophysiol 2012 108 2545 2553 22914655 10.1152/jn.00576.2012 PMC3545184

[B48] Alvarez P Gear RW Green PG Levine JD IB4-saporin attenuates acute and eliminates chronic muscle pain in the rat Exp Neurol 2012 233 859 865 22206923 10.1016/j.expneurol.2011.12.019 PMC3272112

[B49] Hoeger-Bement MK Sluka KA Phosphorylation of CREB and mechanical hyperalgesia is reversed by blockade of the cAMP pathways in a time-dependent manner after repeated intramuscular acid injections J Neurosci 2003 23 5437 5445 12843242 10.1523/JNEUROSCI.23-13-05437.2003 PMC6741249

[B50] Chen WK Liu IY Chang YT Chen YC Chen CC Yen CT Shin HS Chen CC Cav3.2 T-type Ca2+ channel-dependent activtation of ERK in paraventricular thalamus modulates acid-induced chronic muscle pain J Neurosci 2010 30 10360 10368 20685979 10.1523/JNEUROSCI.1041-10.2010 PMC6634662

[B51] Cheng SJ Chen CC Yang HW Chang YT Bai SW Chen CC Yen CT Min MY Role of extracellular signal-regulated kinase in synaptic transmission and plasticity of a nociceptor input on capsular central amygdaloid neurons in normal and acid-induced muscle pain mice J Neurosci 2011 31 2258 2270 21307262 10.1523/JNEUROSCI.5564-10.2011 PMC6633051

[B52] Brederson JD Jarvis MF Honore P Surowy CS Fibromyalgia: mechanism, current treatment and animal models Curr Pharmaceut Biotech 2011 12 1613 1626 10.2174/138920111798357258 21466451

[B53] Chen CC Zimmer A Sun WH Hall J Brownstein MJ Zimmer A A role for ASIC3 in the modulation of high-intensity pain stimuli Proc Natl Acad Sci USA 2002 99 8992 8997 12060708 10.1073/pnas.122245999 PMC124411

[B54] Stirling LC Forlani G Baker MD Wood JN Mattews EA Dickenson AH Nassar MA Nociceptor-specific gene deletion using heterozygous Na_V_1.8-Cre recombinase mice Pain 2005 113 27 36 15621361 10.1016/j.pain.2004.08.015

[B55] Nakamura T Colbert MC Robbins J Neural crest cells retain multipotential characteristics in the developing valves and label the cardiac conduction system Circ Res 2006 98 1547 1554 16709902 10.1161/01.RES.0000227505.19472.69

[B56] Drew LJ Rohrer DK Price MP Blaver KE Cockayne DA Cesare P Wood JN Acid-sensing ion channels ASIC2 and ASIC3 do not contribute to mechanically activated currents in mammalia sensory neurons J Physiol 2004 556 691 710 14990679 10.1113/jphysiol.2003.058693 PMC1664992

